# MiR-20a, a novel promising biomarker to predict prognosis in human cancer: a meta-analysis

**DOI:** 10.1186/s12885-018-4907-3

**Published:** 2018-11-29

**Authors:** Donghua Huang, Yizhong Peng, Kaige Ma, Xiangyu Deng, Lu Tang, Doudou Jing, Zengwu Shao

**Affiliations:** 1 Department of Orthopaedics, Union Hospital, Tongji Medical College, Huazhong University of Science and Technology, Wuhan, 430022 China; 2 Department of Hematology, Union Hospital, Tongji Medical College, Huazhong University of Science and Technology, Wuhan, 430022 China

**Keywords:** miR-20a, Prognosis, Meta-analysis, Cancer

## Abstract

**Background:**

Recently, microRNA-20a (miR-20a) has been reported to influence the clinical features and may have prognostic value in human cancers. The present meta-analysis assessed the prognostic role of miR-20a in various carcinomas.

**Methods:**

Literature searches of seven electronic databases were performed for eligible articles of the prognostic role of miR-20a in human cancers. Hazard ratios (HR) for overall survival (OS), disease free survival (DFS), progression-free survival (PFS) as well as their 95% confidence intervals (95%CIs) were used to assess the influence of miR-20a expression on patient prognosis. Odds ratio (OR) and 95%CIs were applied to evaluate the correlation between miR-20a expression and clinicopathological characteristics.

**Results:**

Based on the OS analyzed by log rank tests, there was a significant association between miR-20a levels and OS by fixed effects model. By subgroup analyses, the significance was also observed in the studies of specimen derived from blood and gastrointestinal cancer group. The independent prognostic role of miR-20a expression for the OS was observed significantly by fixed effects model. In addition, we observed significant association between miR-20a expression levels and DFS of log rank tests, DFS of cox regression. Significant relation of gender/differentiation and the expression level of miR-20a was identified.

**Conclusions:**

Base on the findings, the elevated miR-20a expression level is related to poor prognosis of gastrointestinal cancer patients. As for other types of carcinomas, the results are still not stable and more studies are required to further identify miR-20a prognostic values. In addition, miR-20a expression level is relatively higher in women than that in men, and increased miR-20a expression level is linked to poor tumor differentiation.

## Background

Cancer has become the major social health problem, and it is now the leading cause of mortality worldwide due to its growing incidence each year and poor prognosis. Although new treatment approaches, such as surgery, radiotherapy and chemotherapy, have been elaborately developed, the clinical outcome of carcinomas still remains unsatisfied. One of the reasons is lack of effective biological markers help to define subgroups of patients who might benefit or not benefit from some specific treatments. Therefore, exploring potential diagnostic and prognostic biomarkers for human malignancies to guide clinical decision is crucial and urgent.

MicroRNAs (miRNA) are small noncoding molecules of with a length of approximately 18–24 nucleotides, and can negatively regulate their target genes expression [1, 2]. Many miRNAs have been identified to express abnormally in human malignancies and can play an oncogenic or anti- oncogenic role in tumor biological behaviors [3, 4]. Owing to its stability and detectability in tissues/blood, miRNA is one of the most promising biomarkers for the prognosis of human cancers [5–7].

MiR-20a which is one member of miR-17-92 cluster, has been identified to be closely associated with cancer proliferation [8, 9], invasion [10], metastasis [8, 9, 11] or chemotherapeutic resistance [12, 13] by recent studies. These could be largely attributed to the active biological activities of miR-20a in inference the cellular signal pathways, such as PTEN/PI3K/AKT pathway [14, 15], MAPK1/c-Myc parthway [16], ENH1/Id1 parthway [17], FAS promoter activity [18], TAKI expressions [19], FBXL5/BTG3 signaling [20], the Sonic Hedgehog pathway [21] and etc. Nevertheless, there exist inconsistencies about prognostic accuracy of miR-20a, though numerous studies have identified the associations between miR-20a and various human cancers. Wang et al. [22], Cheng et al. [23], Xu et al. [24] and Reng et al. [25] found that the high expression level of miR-20a was associated with a poor survival rates in cancer patients. But Chang et al. [26], Zhang et al. [27] and Fan et al. [28] explored an anti-tumor effect of miR-20a and patients in their studies benefited from up-regulated miR-20a. Marchini et al. [29], and Xu et al. [30] observed no statistically relationship between expression level of miR-20a and overall survivals of patients. Hence, based on the whole published relevant researches, a systematic analysis was conducted to assess the prognostic efficiency of miR-20a in human cancers as well as the association between miR-20a expression and cancer patients’ clinical characteristics.

## Methods

### Publication selection

An electronic search of PubMed, Web of Science (WOS), Embase in English and VIP, Wanfang, SinoMed and the China National Knowledge Infrastructure (CNKI) in Chinese was applied to select articles using the following keywords: ‘tumor’ or ‘cancer’ or ‘carcinoma’ or ‘neoplasm’ or ‘malignancies’ and ‘miRNA-20a’ or ‘miR-20a’ and ‘prognos*’, ‘surviv*’. We also retrieved articles manually from other sources to complement the results. The search was updated in July 13 2018.

### Eligibility criteria

Studies from the initial researches that satisfy the criteria below were thought to be eligible. (1) studies evaluated the prognostic value of blood or tissue miR-20a level in various human cancers. (2) the relationships between miR-20a expression and patients’ survival were described; (3) Studies have sufficient data to calculate the hazard ratios (HR) and 95%confidence interval (95%CI) for survival rates or odds ratio (OR) and 95%CI for the correlation between miR-20a expression and clinicopathological characteristics. (4) there was no restrictions on the methods of detecting the miR-20a expression levels in the cancer patients by some specific methods, such as qRT-PCR, microarray or etc.

Studies were excluded if (1) patients were of benign tumors. (2) there were notthe primary carcinomas but metastatic carcinomas from other organs. (3) the articles were letters, duplicated publications, reviews or case reports. (4) literatures were published in languages other than English or Chinese.

### Data extraction

To ensure the accuracy of data extraction, two authors (DH and YP) extracted data separately from the eligible studies and inconsistencies were solved by a third senior author (KM). For all enrolled studies, the following information was listed: the first author; year of publication; country; tumor type and clinical stage; number of patients included; the type of specimen; detection methods of miR-20a expression levels; follow-up time; cut-off values; survival analysis and their source of HR; HR for overall survival (OS), disease free survival (DFS), progression-Free-Survival (PFS) and relapse free survival (RFS) as well as their 95%CIs and the quality of study. What’s more, the clinicopathological characteristics of including patients were extracted from some studies which reported the data.

Figures of HR and its corresponding 95% CI of univariate and/or multivariate analyses could be directly obtained from some of studies, whereas others only showed Kaplan-Meier curves without specific data. For these researches, we extracted necessary data from Kaplan-Meier curves by Engauge Digitizer version 9.8 and then input the extracted survival rates at specific time points into the spreadsheet constructed by Tierney et al. [31] to acquire the HR and its corresponding 95%CIs.

### Quality assessment

All the included studies were retrospective and non-randomized studies. We applied the Newcastle-Ottawa Scale (NOS) for evaluating the quality [32]. The NOS scores ranged from 0 to 9, and score more than 6 was regarded as high quality. Three authors (DH, YP and KM) assess the quality independently and any disagreement was resolved by discussion.

### Statistical analysis

The PRISMA checklists and their guidelines were strictly followed during the whole procedure of the study [33, 34]. The meta-analysis was conducted with software version 14.0 (Stata Corporation, College Station, TX, USA). The pooled HRs and their 95%CIs were used to assess the impact of miR-20a expression levels on clinical prognosis for OS, DFS and PFS. The adjusted HRs (95 %CIs) for OS and DFS were also calculated using data extracted from the cox regression. HRs larger than 1 denoted poorer prognosis in patients with increased miR-20a expression. The fixed effects model and the random pooling model were both used in the analyses. P less than 0.05 or the 95%CI did not overlap with 1 indicated statistically significant. The heterogeneity among studies was calculated by the Chi square-based *Q* test and I^2^ statistics. *P* value less than 0.10 for the *Q* test or I^2^ larger than 50% was considered as significant heterogeneity. Subgroup analyses stratified by population (Chinese and Italian), sample size (< 150 and ≥ 150), NOS scores (< 8 and ≥ 8), specimen (blood and tissues) and tumor category (gastrointestinal cancer and non-gastrointestinal cancer) were carried out. The sensitivity analysis also managed to assess the stability of the results by omitting each study in turn. Publication bias was estimated by visually evaluating the asymmetry of the funnel plot. What’s more, Egger’s linear regression test and Begg’s funnel plot test were applied to offer quantitative evidence of publication bias. The odds ratios (ORs) and corresponding 95%CIs were also computed to detect the relation of miR-20a expression to clinicopathological characteristics. All *P* values were two tailed.

## Results

### Characteristics of the enrolled studies

**Fig. 1:**
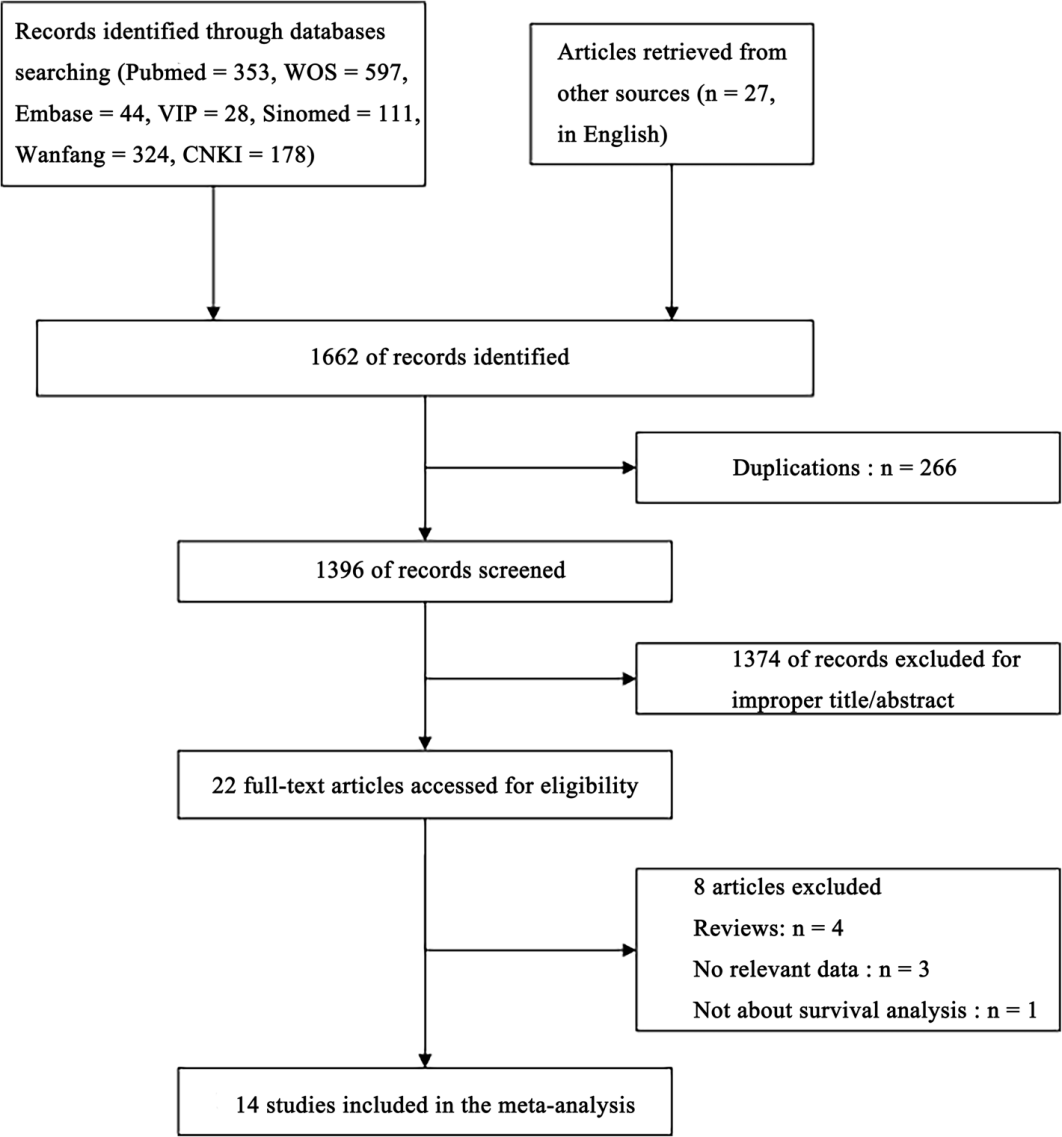
The flow chart of the meta-analysis

The article retrieval strategy was shown in Fig. 1. The initial search identified a total of 1662 papers, 266 of which were removed due to duplications. Titles/abstracts of there maining 1396 publications were reviewed, 1374 being excluded and leaving 22 as candidate literatures. After a full text evaluation, a total of 14 studies were finally included [22–30, 35–39].

The articles were published between 2011 and 2018. The total number of subjects included in the current meta-analysis was 1822, and the sample size ranged from 52 to 544 with a mean value of 130.1. Twelve enrolled studies were carried out in China, and the other two studies were conducted in Europe. There were eight types of cancer in the included papers, with three studies for squamous cell carcinoma, three studies for gastric cancer, two studies for NSCLC, two studies for ovarian cancer, one study for colorectal cancer, one study for glioblastoma, one study for multiple myeloma and one study for hepatocellular carcinoma. There were 10, 4, 2, 1 studies containing HR and its corresponding 95% CI for OS, DFS, PFS and RFS, respectively. Thirteen studies measured the miR-20a expression level by qRT-PCR (Real-time Polymerase Chain Reaction), while two studies also applied the methods of miRNA array besides qRT-PCR and one study used the nCounter Human v2 miRNA Expression Assay described in its article [38]. The main information of the included studies was performed in Table 1.

**Table 1:** Characteristics of studies included in the meta-analysis

NO	Study	Year	Race	Tumor type	Clinical stage of tumor	NO(high/low)	Specimen	Detection method	Cut-off value	Outcome	Survival analyses and their source of HR	Follow-up time	NOS
1	Wang et al.	2012	China	GC	TNM I-IV	OS: 65(34/31)	blood	*qRT-PCR*	median value	OS	OS: univariate(reported), multivariate(reported)	3 years	8
2	Chang et al.	2013	China	OSCC	TNM I-IV	OS: 98(71/27)	tissues	qRT-PCR	#	OS	OS: univariate(K-M Curve)	84 months	8
3	Cheng et al.	2016	China	CRC	TNM I-IV	OS, DFS: 544(407/137)	tissues	qRT-PCR	N/D	OS, DFS	OS, DFS: univariate(K-M Curve), multivariate(reported)	about 110 months	8
4	Fan et al.	2013	China	HCC	tumor stage I-III	OS, RFS: 100(50/50)	tissues	qRT-PCR	N/D	OS, RFS	OS, RFS: univariate(reported), multivariate(reported)	100 months	7
5	Gao et al.	2012	China	MM	ISS: I-III, D-S: I-III	PFS: 85(43/42)	BM	qRT-PCR	N/D	PFS	PFS: univariate(K-M Curve)	35 months	6
6	Huang et al.	2014	China	GC	N/D	OS: 82(41/41)	blood	qRT-PCR	N/D	OS	OS: univariate(N/D)	about 20 months	6
7	Marchini et al.	2011	Italy	EOC	Figo I substage A-C	OS, PFS: 89(N/D)	tissues	Microarray and qRT-PCR	&	OS, PFS	OS, PFS: univariate(reported), multivariate(reported)	9 years	8
8	Sanfiorenzo et al.	2012	France	NSCLC	TNM IA-B, IIA-B, IIIA	DFS: 52(N/D)	tissues and blood	qRT-PCR	median	DFS	DFS: univariate(K-M Curve), multivariate(reported)	60 months	7
9	Xu et al.	2013	China	ESCC	Clinical stage I-IV	OS: 105(54/51)	tissues	qRT-PCR	N/D	OS	OS: univariate(K-M Curve)	about 55 months	8
10	Xu et al.	2018	China	NSCLC	TNM I-III	OS, DFS: 196(N/D)	blood	qRT-PCR	median	OS, DFS	OS, DFS: univariate(reported), multivariate(reported)	about 100 months	8
11	Yang et al.	2016	China	GC	TNM I-IV	OS: 55(27/28)	blood	Microarray and qRT-PCR	median	OS	OS: univariate(K-M Curve)	about 35 months	8
12	Zhang et al.	2015	China	CSCC	TNM I-III	OS: 152(54/98)	tissues	qRT-PCR	N/D	OS	OS: univariate(K-M Curve), multivariate(reported)	60 months	6
13	Zhao et al.	2017	China	glioblastoma	N/D	OS, DFS: 106(N/D)	blood	the nCounter Human v2 miRNA Expression Assay	N/D	OS, DFS	OS, DFS:univariate(K-M Curve), multivariate(reported)	2 years	7
14	Reng et al.	2016	China	OSCA	Figo I-IV	OS: 93(71/22)	tissues	qRT-PCR	N/D	OS	OS: univariate(K-M Curve)	about 45 months	5

Abbreviations: *BM* Bone marrow*, CRC* Colorectal cancer*, CSCC* Cutaneous squamous cell carcinoma, *DFS Disease-free survival, D-S* Durie-Salmon*, EOC* Epithelial ovarian cancer*, ESCC* Esophageal squamous cell carcinoma, *FIGO* The International Federation of Gynecology and Obstetrics*, GC* Gastric cancer*, HCC* Hepatocellular carcinoma*, MM* Multiple myeloma*, NOS* Newcastle-Ottawa scale scores*, NSCLC* Non-small cell lung carcinoma*, N/D* Not described*, OS* Overall survival*, OSCA* Ovarian serous cystadenocarcinoma*, OSCC* Oral squamous cell carcinoma*, PFS* Progression-free survival*, qRT-PCR* Quantitative Real-time PCR*, RFS* Recurrence-free survival;#, the article difined the high miR-20a expression level as multiple of change of larger than 0.24 and low as multiple of change less than or equal to 0.24; &, The Contal and O’Quigley method was applied (SAS macro was provided by Mandrekar and colleagues) to choose a cutoff value

### The association between miR-20a expression levels and overall survival (OS)

**Fig. 2:**
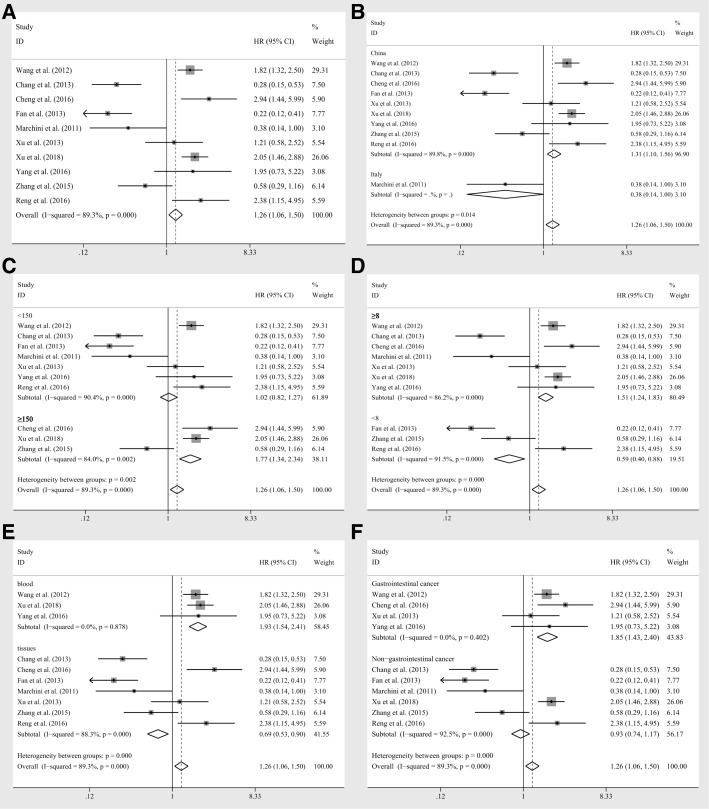
The association between miR-20a expression levels and **a** overall survival, subgroup analyses of **b** population (Chinese and Italian) **c** sample sizes (≥150 and < 150), **d** NOS scores (≥8 and < 8), **e** specimen (blood and tissues), **f** tumor category (gastrointestinal cancer and non-gastrointestinal cancer) by fixed effects model

Ten enrolled studies including 1497 patients investigated the relation of miR-20a expression levels to the prognostic parameters (OS) using log rank tests, resulting in the univariate data. Generally, there was a significant association between miR-20a levels and OS (HR = 1.26, CI: 1.06–1.50, Fig. 2a), however, a significant heterogeneity was observed among the researches (I^2^ = 89.30%, *P* < 0.10, Table 2). Whereby, the random pooling model was applied in succession and the significance was vanished (HR = 0.99, CI: 0.56–1.75, Table 2), indicating that the heterogeneity influenced the results significantly. Then subgroup analyses were conducted by factors including population (Chinese and Italian), sample size (≥150 and < 150), NOS scores (≥8 and < 8), specimen (blood and tissues) and tumor category (gastrointestinal cancer and non-gastrointestinal cancer), so as to diminish the heterogeneity. As a consequence, the heterogeneity was controlled successfully in the group with specimen derived from blood (I^2^ = 0.00%, *P* = 0.878, Table 2) and the corresponding significance was obvious (HR = 1.93, CI: 1.54–2.41, Fig. 2e). Also, the gastrointestinal cancer group revealed eliminated heterogeneity as well (I^2^ = 0.00%, *P* = 0.402, Fig. 2f), and the relation of miR-20a levels to OS was also significant (HR = 1.85, CI: 1.43–2.40, Fig. 2f). Moreover, significant associations were observed between miR-20a expression levels and OS in the studies with Chinese samples (HR = 1.31, CI: 1.10–1.56, Fig. 2b), sample size greater than or equal to 150 (HR = 1.77, CI: 1.34–2.34, Fig. 2c), NOS scores greater than or equal to 8 (HR = 1.51, CI: 1.24–1.83, Fig. 2d) or less than 8 (HR = 0.59, CI: 0.40–0.88, Fig. 2d) and specimen derived from tissues (HR = 0.69, CI: 0.53–0.90, Fig. 2e) by fixed pooling model, while there were no significances identified in these groups, when the random pooling model was applied (Table 2). And the heterogeneities within the subgroups were still significant, except for the groups of gastrointestinal cancer as well as samples derived from tissues.

**Table 2:** Association between miR-20a expression levels and overall survivals

	No. of studies	No. of patients	Pooled HR(95%CI)		Meta regression	Heterogeneity	
			Fixed	Random	*p*-value	I^2^	*p*-value
Overall	10	1497	1.26(1.06,1.50)	0.99(0.56,1.75)		89.30%	0.000
Population					0.894		
Chinese	9	1408	1.31(1.10,1.56)	1.09(0.60,1.96)		89.80%	0.000
Italian	1	89	0.38(0.14,1.00)	0.38(0.14,1.00)		–	–
Sample Size					0.405		
≥ 150	3	892	1.77(1.34,2.34)	1.54(0.67,3.54)		84.00%	0.000
< 150	7	605	1.02(0.82,1.27)	0.82(0.37,1.79)		90.40%	0.000
NOS Scores					0.829		
≥ 8	7	1152	1.51(1.24,1.83)	1.19(0.67,2.13)		86.20%	0.000
< 8	3	345	0.59(0.40,0.88)	0.67(0.17,2.57)		91.50%	0.000
Specimen					0.621		
blood	3	316	1.93(1.54,2.41)	1.93(1.54,2.41)		0.00%	0.878
tissues	5	1181	0.69(0.53,0.90)	0.73(0.33,1.62)		88.30%	0.000
Tumor Category					0.350		
Gastrointestinal cancer	4	769	1.85(1.43,2.40)	1.85(1.43,2.40)		0.00%	0.402
Non-gastrointestinal cancer	6	728	0.93(0.74,1.17)	0.65(0.26,1.61)		92.50%	0.000

Abbreviations: *95%CI* 95% confidence interval, *Fixed* Fixed effects model, *HR* hazard ratio, *NOS* Newcastle-Ottawa scale scores, *Random* Random pooling model

**Fig. 3:**
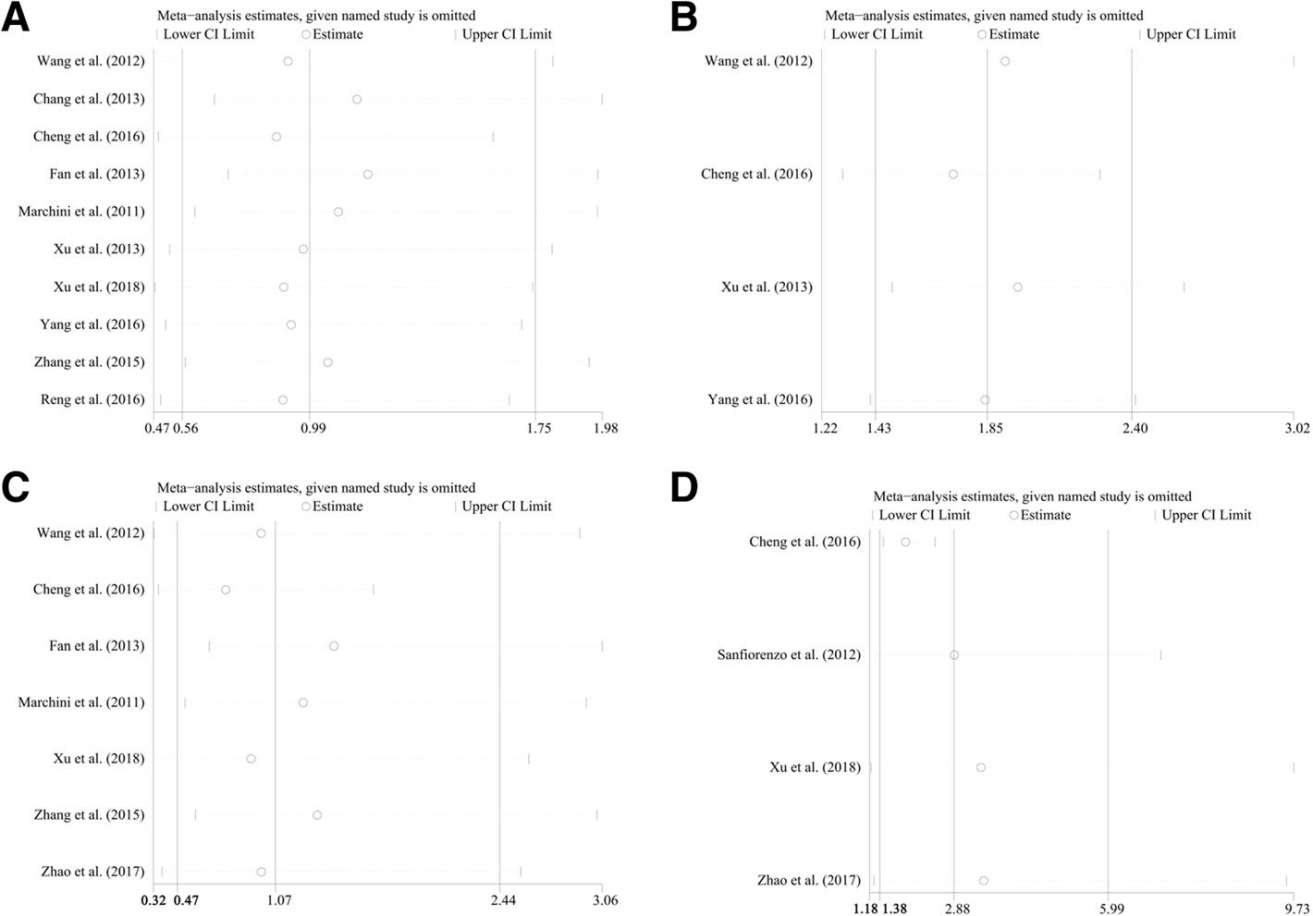
Sensitivity analyses for HRs of overall survivals extracted from **a** log tests and **c** coxmultivariate regression; and sensitivity analyses for **b** gastrointestinal group and **d** DFS derived from cox multivariate regression

Based on the results above, meta regression was further performed, but there was no significant contribution identified to greatly influence the variation of HRs (*p* = 0.894 for population, *p* = 0.405 for sample size, *p* = 0.829 for NOS scores, *p* = 0.621 for specimen, *p* = 0.350 for tumor category, respectively, Table 2). Moreover, the sensitivity analysis was performed and no studies seemed to have great impacts on the significance of the results (Fig. 3a). In addition, funnel plots, Begg’s rank correlation and Egger’s weighted regression method were implemented to evaluate the publication bias. Though the efficacy of these methods might be limited due to the insufficient studies amount, we still chose those methods for lack of alternatives. The funnel plot of all seven studies reported symmetric and the Begg’s, Egger’s tests revealed no significant publication bias (*P* = 0.721, *P* = 0.213, respectively). The sensitivity analysis within the gastrointestinal cancer group also revealed that no studies could significantly impacted the results, indicating the stableness and reliability of the results (Fig. 3b).

### The independent role of miR-20a expression level as prognostic indicator

**Fig. 4:**
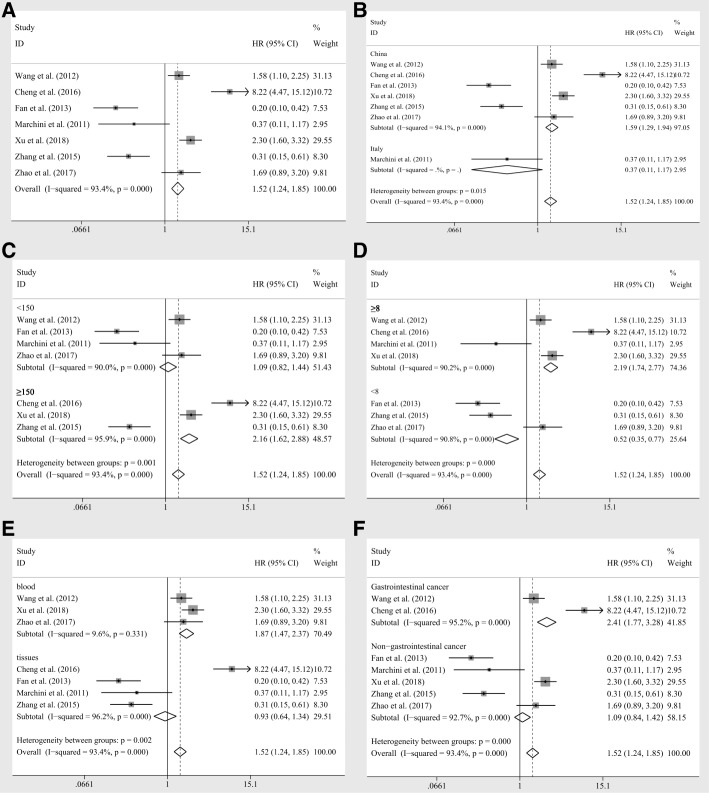
The independent role of miR-20a as a prognostic detector for the overall survivals in patients of carcinoma, **a** overall survivals, subgroup analyses of **b** population (Chinese and Italian) **c** sample sizes (≥150 and < 150), **d** NOS scores (≥8 and < 8), **e** specimen (blood and tissues), **f** tumor category (gastrointestinal cancer and non-gastrointestinal cancer) by fixed effects model

Seven researches containing 1252 patients implemented the cox multivariate regression to evaluate the prognostic value of miR-20a expression levels in cancer patients, adjusting other factors. The significant relation of miR-20a expression to the OS (HR = 1.52, CI: 1.24–1.85, Fig. 4a) was observed by fixed pooling model. However, the significance was vanished by random pooling model (HR = 1.07, CI: 0.47–2.44, Table 3) and the heterogeneity was relatively obvious (I^2^ = 93.40%, *P* < 0.10, Table 3). Similarly, to reduce heterogeneity, subgroup analyses were applied and the homogeneity was reached within the studies of samples derived from blood (I^2^ = 9.60%, *P* = 0.331, Table 3). And the significant association was identified between miR-20a expression levels and OS within the group of samples derived from blood (HR = 1.87, CI: 1.47–2.37, Fig. 4e). In addition, the significant relations of miR-20a expression levels to OS were also recognized within the studies of Chinese samples (HR = 1.59, CI: 1.29–1.94, Fig. 4b), sample size greater than or equal to 150 (HR = 2.16, CI: 1.62–2.88, Fig. 4c), NOS scores greater than or equal to 8 (HR = 2.19, CI: 1.74–2.77, Fig. 4d) or less than 8 (HR = 0.52, CI: 0.35–0.77, Fig. 4d) and the patients of gastrointestinal cancer (HR = 2.41, CI: 1.77–3.28, Fig. 4f) by fixed effects model, which turned out to be of no significance within those subgroups by random pooling model (Table 3). Sensitivity analyses revealed no studies had significant impacts on the results (Fig. 3c). Furthermore, no obvious publication bias was identified among the four studies (*P* = 0.230 for Begg’s test and *P* = 0.287 for Egger’s test, respectively). Moreover, meta regression was further performed to identify the underlying factors contributing to the variation of HRs. As a result, it suggested that nearly all of the subgroup factors except NOS scores had contributed to the between-study variance (*p* = 0.020 for population, *p* = 0.011 for sample size, *p* = 0.107 for NOS scores, *p* = 0.040 for specimen, *p* = 0.012 for tumor category, respectively). Normalizing all the significant factors observed, the estimate of between-study variance, Tau-squared (tau^2^), plummeted from 1.1191 to 0, indicating that these factors completely explained the between-study variance. Also, the residual heterogeneity was diminished (I^2^ = 0.00%).

**Table 3:** Meta analysis of miR-20a as an independent prognostic indicator for patients of various caricinomas

	No. of studies	No. of patients	Pooled HR(95%CI)		Meta regression	Heterogeneity	
			Fixed	Random	*p*-value	I^2^	*p*-value
Overall	7	1252	1.52(1.24,1.85)	1.07(0.47,2.44)		93.40%	0.000
Population					0.020		
Chinese	6	1163	1.59(1.29,1.94)	1.24(0.52,2.96)		94.10%	0.000
Italian	1	89	0.37(0.11,1.17)	0.37(0.11,1.17)		–	–
Sample Size					0.011		
≥ 150	3	892	2.16(1.62,2.88)	1.82(0.38,8.67)		95.90%	0.000
< 150	4	360	1.09(0.82,1.44)	0.70(0.25,1.96)		90.00%	0.000
NOS Scores					0.107		
≥ 8	4	894	2.19(1.74,2.77)	2.03(0.89,4.63)		90.20%	0.000
< 8	3	358	0.52(0.35,0.77)	0.48(0.13,1.76)		90.80%	0.000
Specimen					0.040		
Blood	3	367	1.87(1.47,2.37)	1.86(1.45,2.40)		9.60%	0.331
Tissues	4	885	0.93(0.64,1.34)	0.67(0.10,4.60)		96.20%	0.000
Tumor Category					0.012		
Gastrointestinal cancer	2	609	2.41(1.77,3.28)	3.53(0.70,17.81)		95.20%	0.000
Non-gastrointestinal cancer	5	643	1.09(0.84,1.42)	0.64(0.47,2.44)		92.70%	0.000

Abbreviations: *95%CI* 95% confidence interval, *Fixed* Fixed effects model, *HR* Hazard ratio, *NOS* Newcastle-Ottawa scale scores, *Random* Random pooling model

### The relation of miR-20a expression levels to DFS and PFS

**Fig. 5:**
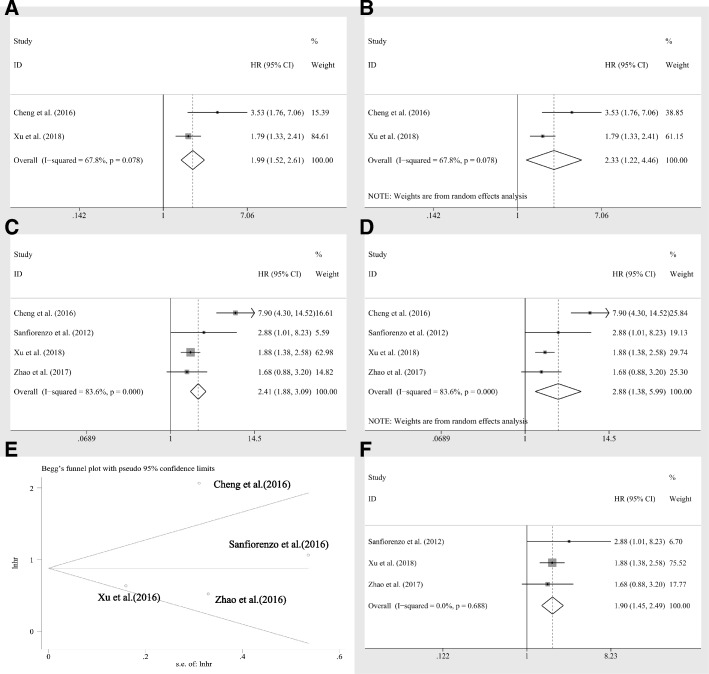
The association between miR-20a expression levels and DFS extracted from log rank tests by **a** fixed effects model and **b** random pooling model, cox regression by **c** fixed effects model and **d** random pooling model, **f** cox regression by fixed effects model after removing the outlier; **e** publication bias evaluation for DFS

Four studies reported disease-free survival (DFS), of which two studies applied only log rank tests, while others also utilized cox multivariate regression. Thus, after pooling the HR, we observed significant association between miR-20a expression levels and DFS of log rank tests (HR = 1.99, CI: 1.52–2.61, Fig. 5a), DFS of cox regression (HR = 2,41, CI: 1.88–3.09, Fig. 5c) by fixed effects model. However, the heterogeneities were rather obvious (I^2^ = 67.80%, *P* = 0.078, Fig. 5a; I^2^ = 83.6%, *P* = 0.000, respectively Fig. 5c). Nevertheless, the significance for data extracted from log rank tests and cox regression still existed by random pooling model (HR = 2.33, CI: 1.22–4.46, Fig. 5b; HR = 2.88, CI: 1.38–5.99, Fig. 5d), indicating the stability of the results. Furthermore, owing to limited number of statistics from log rank tests, the sensitivity analysis was only applied to the analyses with data extracted from cox regression, revealing that no studies had great impacts on the results (Fig. 3d). However, the investigation of publication bias identified an outlier (Fig. 5e), Cheng et al. [23] After removing this study, the heterogeneity was completely eliminated (I^2^ = 0.00%, *P* = 0.688, Figure5F) and the significance of association between miR-20a expression levels and DFS was not altered (HR = 1.90, CI: 1.45–2.49, Fig. 5f).There were only two studies containing 174 patients revealed the available PFS statistics. However, the prognostic value of miR-20a expression levels to PFS were completely different (Gao et al. [39] HR = 2.25, CI 1.18–4.30; Marchini et al. [29] HR = 0.36, CI 0.16–0.80). Due to insufficient data, pooling effects would be lack of efficiency and the heterogeneity was significant (I^2^ = 91.8%, *P* < 0.10). Thus, more relevant studies reporting the prognostic effects of miR-20a to PFS are required to perform the analysis.

### Correlations between miR-20a levels and clinicopathological features among various carcinomas

**Fig. 6:**
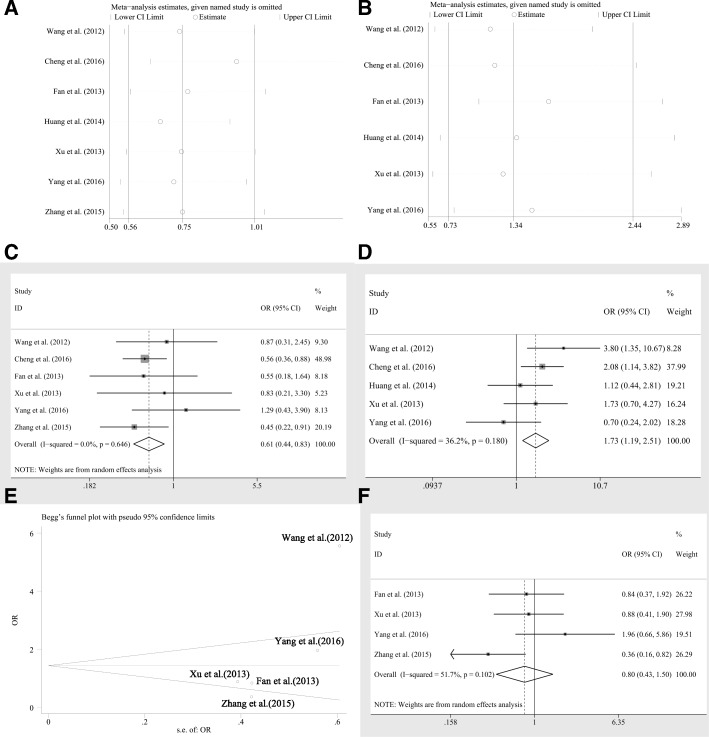
Sensitivity analyses for the pooling relation of miR-20a expression levels to clinicopathological characteristics, such as **a** gender, **b** tumor differentiation degree; the association between miR-20a expression levels and **c** gender, **d** tumor differentiation degree or **f** TNM stages without the outlier; **e** publication bias evaluation for the studies reporting TNM stages

There are seven articles containing 1103 patients of various carcinomas investigated the association between miR-20a expression levels and different clinic characteristics. As shown in Table 4, there were significant relations observed among gender (OR = 0.69, CI: 0.51–0.92), differentiation (OR = 1.50, CI: 1.06–2.13) by fixed effects model to the expression level of miR-20a. However, the significance was altered by random pooling model. Moreover, there were no significance identified in the association between age (OR = 1.44, CI: 0.81–2.57), tumor sizes (OR = 0.77, CI: 0.44–1.36), lymph node metastasis (OR = 1.03, CI = 0.48–2.21) or TNM stage (OR = 1.15, CI: 0.51–2.58) and the expression level of miR-20a. The heterogeneity was absent in the analysis of age (I^2^ = 0.00%, *P* = 0.373) and moderate in the analysis of gender (I^2^ = 30.70%, *P* = 0.194) but obvious in the analyses of tumor sizes (I^2^ = 49.20%, *P* = 0.116), lymph node metastasis (I^2^ = 54.30%, *P* = 0.112), TNM stage (I^2^ = 74.40%, *P* = 0.004) and differentiation (I^2^ = 59.10%, *P* = 0.032). Sensitivity analysis and investigation of publication bias were applied to each clinic characteristic analysis. The sensitivity analysis of the gender identified a study, Huang et al. [35], which had significant impact on the pooling results (Fig. 6a). After remove the outlier, the heterogeneity was greatly decreased from 30.70 to 0.00%, and also there was a significant association between miR-20a expression and gender (HRs = 0.61, CI: 0.45–0.83, Fig. 6c). In addition, the sensitivity analysis recognized another outlier in the characteristics analysis of differentiation, which was Fan et al. [28] (Fig. 6b). The removal of Fan et al. did diminish the heterogeneity (I^2^ = 36.20%, *P* = 0.180, Fig. 6d), and a significant relation was identified of miR-20a expression levels to the degrees of differentiation (HRs = 1.73, CI: 1.19–2.51, Fig. 6d). Moreover, the publication bias was identified in the analysis of TNM stages (*P* = 0.086 for Begg test, *P* = 0.059 for Egger test, respectively). By the Begg’s plot (Fig. 6e), we identified the outlier, Wang et al. [22] The heterogeneity was reduced in an extent (I^2^ = 51.7%, *P* = 0.102), but the pooling result was not significantly altered (HRs = 0.80, CI: 0.43–1.50, Fig. 6f).

**Table 4:** Overall analysis of miR-20a expression association with clinicopathological characteristics

Clinicopathological parameters	No. of studies	No. of patients	Pooled OR (95%CI)		Heterogeneity	
			Fixed	Random	I^2^	*p*-value
Gender (male vs. female)	7	1103	0.69(0.51,0.92)	0.74(0.50,1.09)	30.70%	0.194
Age (≥60 vs < 60 years)	3	202	1.44(0.81,2.55)	1.44(0.81,2.57)	0.00%	0.373
Tumor Size (≥5 vs < 5 cm)	4	422	0.76(0.51,1.12)	0.77(0.44,1.36)	49.20%	0.116
Lymph node metastasis (absent vs.present)	3	260	1.11(0.68,1.81)	1.03(0.48,2.21)	54.30%	0.112
TNM stage(III + IV vs. I + II)	5	467	0.96(0.66,1.41)	1.15(0.51,2.58)	74.40%	0.004
Differentiation (poor vs. others)	6	951	1.50(1.06,2.13)	1.34(0.73,2.44)	59.10%	0.032

Abbreviations: *95%CI* 95% confidence interval, *Fixed* Fixed effects model, *OR* Odds ratio, *Random* Random pooling model

## Discussion

MiR-20a, which has been detected to be aberrantly expressed in many malignancies, may play as a novel biomolecule in tumor progression [40–42]. Several genes or signal pathways have been discovered to be targeted by miR-20a in tumor biological behavior, such as KIF26B in osteosarcoma [43], RB1CC1/FIP200 in breast cancer [44], LIMK1 in anaplastic thyroid cancer [45], ABL2 in prostate cancer [42] and etc. There are three systematic reviews (Zheng et al., Li et al., Troiano et al.) summarizing the prognostic value of miR-20a expression in specific type of tumor recently: Zheng et al. [46] and Li et al. [47] generalized that the up-regulated expression of miR-20a was correlated with a poor prognosis in gastrointestinal cancer and cervical cancer, respectively. Nevertheless, Troiano et al. [48] came to an opposite conclusion in oral squamous cell carcinoma against the former two articles. A number of studies have been carried out to illustrate the prognostic role of miR-20a in cancer, but the underlying value of miR-20a for survival rates in various cancer patients remains unclear.

The current meta-analysis intended to explore the association between the expression level of miR-20a and human cancer prognosis. To our knowledge, this is the most comprehensive meta-analysis providing insights into the clinical value of miR-20a in various types of human cancers currently. Fourteen papers including 1822 patients were recruited in this meta-analysis. Ten studies containing 1497 patients reported the statistics of OS as a result of log rank tests. By the pooling strategy, we identified that the elevated miR-20a expression was linked to poor prognosis of cancer patients. Then subgroup analyses were implemented to eliminate the potential sources of heterogeneity. Consequently, the homogeneity was reached within the groups of gastrointestinal cancer and samples derived from blood, and the OS of gastrointestinal cancer group was found to be greatly associated with the miR-20a expression levels. In addition, seven articles including 1252 patients contained the data of HRs derived from cox multivariate regression of survival analysis. The cox regression [49] has been proved to be effective in the survival analysis, because it evaluates the contribution of each factor independently by adjusting others. Thus, the results always indicate the independent effects of each factor on the clinic outcome. However, subgroup analyses found that the significance might be vanished in gastrointestinal cancer group when the pooling strategy was changed from fixed effects model to random pooling model. As shown in Table 3, the subgroup of gastrointestinal cancer recruited only two studies and the results might not be reliable. In addition, by retrieving the studies, we found that the relation of miR-20a levels to OS was both significant and consistent (Wang et al. [22] HR = 1.58, CI 1.10–2.25; Cheng et al. [23] HR = 8.22, CI 4.47–15.12), which means that it is still safe to draw the conclusion that overexpression of miR-20a is linked to poor prognosis of gastrointestinal cancer patients. Moreover, meta regression illustrated that the contribution of various factors included population, sample size, specimen and tumor category. By adjusting all the identified factors, the between-study variance was completely explained and furthermore, the remaining heterogeneity was diminished.

Upregulation of miR-20a has been found to inhibit the proliferation, invasion and migration of cancer cells [16, 50, 51]. Whereby, overexpression of miR-20a has been reported to promote migration and invasion of various cancers [52–54]. The regulation of miR-20a to cancer cells verified from different cancers, and even for the same kind of carcinoma such as breast cancer, the results are controversial [16, 52]. But the result is consistent among gastrointestinal cancers, such as colorectal cancer [55], gastric cancer [56] and etc. Though the mechanism of how miR-20a induces unfavorable outcome of gastrointestinal cancers is still not clarified, there are several potential explanations. It has been reported that miR-20a/LRIG1 axis might regulate gastric cancer drug resistance through EGFR-mediated PI3K/AKT and MAPK/ERK signaling [57]. Also, miR-20a has been found to be able to repressed the expression of cylindromatosis, leading to activation of the NF-κB pathway and the downstream targets, livin and survivin, which potentially induced GC chemoresistance [58]. In addition, knockdown of miR-20a enhanced sensitivity of colorectal cancer cells to cisplatin through the ROS/ASK1/JNK pathway [59]. Besides, overexpression of miR-20a could induce gastric cancer progression by miR-20a (miR-17)-FBXO31-CyclinD1 pathway [60]. Based on our findings, we could conclude that the elevated miR-20a expression level is associated with poor prognosis of gastrointestinal cancer patients. But for the other types of carcinomas, the results were still not stable and more studies including normalized research conditions (such as specimens, miRNA cut-off values, miRNA detection methods, etc.) were required to further identify miR-20a prognostic value.

As for the clinical features, seven articles enrolled in our analysis including 1103 patients have evaluated the relation of miR-20a to the clinic characteristics. Significant association between miR-20a expression levels and gender or differentiation by fixed effects model was identified. However, due to rather high heterogeneity, the results were not stable and the significances were vanished by random pooling model. Appling sensitivity analyses, we identified two studies (Huang et al. [35] and Fan et al. [28]) that had great impact on the results for the gender and differentiation groups, respectively. After removing them, the heterogeneity completely disappeared for gender group and largely reduced for differentiation group. Moreover,the association between miR-20a expression levels and gender or differentiation was significantly recognized and the results were stable and reliable. As there was still significant heterogeneity existing within other groups that we could not identify the specific sources, further relevant researches were demanded to enrich the results and improve the reliability. Based on the findings, it suggested that women were more likely to develop elevated miR-20a expression, and increased miR-20a expression levels were linked to poor tumor differentiation.

A few limits shall be claimed in this analysis. First of all, the papers language was restricted to English and Chinese and may cause the bias due to lack of other populations. Second, the HRs and its corresponding 95%CI of Yang et al. [37] extracted by the Kaplan-Meier Curves with Engauge Digitizer 9.8 and calculated in the spreadsheet calculator designed by Tierney JF et al. [31] was not consistent to the significance claimed in the original article. Three independent authors (XD, KM and LT) had extracted the data from Yang et al. [37] for several times using the methods described above whose accuracy had been proved by many researches [61–63]. The extracted statistics were always harmonious. But they were different from the significance of the original survival curves. The bias demanded prevention by better precise data extracting methods or improving qualities of the recruited studies. Third, the cutoff values of the expression levels of miR-20a were not precisely acknowledged among some studies included. Fourth, the number of recruited studies was relatively insufficient. More associated researches should be performed and enrolled for this analysis, so as to improve the stability and reliability of the findings.

To enlarge the enrolled studies for the meta-analysis, research checklist for cohort study on http://www.equator-network.org/ is recommended to perform further researches on prognostic values and clinical correlation of certain biomarkers for a specific cancer, then these researches can conform with the inclusion criteria. Also, cut-off value that defines high/low expression of biomarkers (RNAs or genes) should be clearly demonstrated and unified. Besides, the HRs and confidence intervals of log rank test or cox regression should be presented in the form of specific figures, otherwise, the required figures can only be extracted by the Kaplan-Meier Curves with methods described previously, which may induce potential bias.

## Conclusions

Base on the findings, we conclude that the elevated miR-20a expression level is related to poor prognosis of gastrointestinal cancer patients. As for other types of carcinomas, the results are still not stable and more studies including normalized research conditions are required to further identify miR-20a prognostic values. In addition, miR-20a expression level is relatively higher in women than that in men, and increased miR-20a expression level is linked to poor differentiation.

## Abbreviations

95%CIs: 95% confidence intervals
CNKI: The China National Knowledge Infrastructure
DFS: Disease-free survival
HR: Hazard ratio
miRNAs: microRNAs
NOS: The Newcastle-Ottawa Scale
OS: Overall survival
PFS: Progression-free survival
qRT-PCR: Real-time Polymerase Chain Reaction
RFS: Recurrence-free survival;tau^2^, tau-squared
WOS: Web of Science
